# Computer tool with intelligent behavior for the optimal preliminary design in non-braced structural steel frame

**DOI:** 10.1016/j.heliyon.2022.e09260

**Published:** 2022-04-09

**Authors:** Edwin P. Duque, Daniel Villarreal, Henrry Rojas A

**Affiliations:** a Department of Civil Engineering, Technical University of Loja, San Cayetano Alto, Paris Street, Loja 110150, Province of Loja, Ecuador; b Research Group in Seismic Engineering and Seismology of the Technical University of Loja, San Cayetano Alto, Paris Street, Loja 110150, Province of Loja, Ecuador

**Keywords:** Optimization, Structural analysis, Artificial intelligence, Steel structures

## Abstract

The optimization of civil structures is a technique whose purpose is to efficiently use the materials that make up the structural systems based on previously established restrictions and objectives. The use and development of these techniques has been closely linked to technological advance since, through the use of computer equipment, complex mathematical models can be solved with low cost and time. This article presents OPS Design v2.0, a computer tool that allows obtaining a preliminary optimal distribution of metallic structural profiles in a Non-Braced Frame System (OMF: Ordinary Moment Frame). The optimization model implemented in OPS Design v2.0 seeks to minimize the number of different profiles and the structure's own weight in order to reduce the construction complexity and the weight per linear meter (costs in quantities of material). To evaluate its effectiveness, a case study was developed where it was concluded that the designs produced by the application are more efficient than those obtained by commercial tools, thus reducing the computational expense and time used by designers in iterative processes that are carried out in the initial phases project.

## 1 Introduction

The analysis and sizing of a steel structure involves an iterative process in which the structural engineer offers a design that analyzes and checks with stablished norms [1]. In this process, it is implied the search for an optimal solution, that is, fulfilling the security conditions at a bottom cost [2]. In the engineering practice, optimization matters are characterized by the number of decisions and criteria to take into consideration from a multifactorial point of view, and therefore it is natural that optimization techniques based on iterative, combinatorial, or heuristic methods are for emulated. Each optimization method has different properties suitable for different types of problems [3]. An ever-increasing body of literatures shows that the optimum design of steel structures is one of the most common structural problems.

Ha [4] presented an analytical optimization method for the calculation of the minimum cant in type I non-compact beams. His proposal can be compared with a 1969 job developed by Holt and Heithecker, which consisted on the optimal individual design of non-rigid beams, concluding improvements on topics like simplicity and results precision. Changizi & Jalalpour [5] proposed a computational methodology for stress-based topology optimization of steel frame structures. They used Quantile regression to express cross-sectional properties analytically as functions of member cross-sectional area. In addition, their algorithm used the von Mises yield criterion to control the maximum stresses in frames directly. Luévanos et al. [6], use numeric experiments for the optimization, although their approach was on reinforced steel beams, the main objective was to minimize weight and thereby, costs under certain restrictions of deterministic behavior, expressed on mathematical terms. In their study obtaining an optimal design of a beam type element and suggests future research guidelines for steel elements and complete structures. In general, there are more studies, like Mei & Wang [7] show in their article, with different methodologies and approaches.

Since optimization is a process that involves the management of thousands of data, there exist concerns about computational efficiency. For this reason, different researchers like M. Papadrakakis et al. [8] have studied the efficiency of several optimization methods based on mathematical programming and evolutionary algorithms. However, there are alternative methodologies, which use Artificial Intelligence techniques.

Artificial Intelligence is considered a branch of computer science. The term Artificial Intelligence (AI) was used for the first time in 1956, during a conference at Dartmouth College [9]. The main objective of this discipline is to discover how to emulate and perform some of the intelligent functions of the human brain. Nevertheless, there are more definitions, which depend on the perspective of the AI application. AI is a far-reaching and cross-frontier subject applied in many fields, including civil engineering [10].

In this regard Papadrakakis et al. [11] developed an applications of AI to solve optimization problems. They studied the efficiency of combinatorial optimization methods, and they complemented their research with the use of a Neural Network model to replace the structural analysis process. Currently, Neural Network (NN) is a popular method to solve structural optimization problems [12]. It is a powerful tool for data modeling that is capable to detect and represent complex input/output relationships. NN requires less computational effort to produce precise results. Gholizadeh and Mahmmadi [13] proposed a methodology based on the use of NN, the one that was tested with two flat frames in steel of three and ten floors respectively. It is convenient to highlight that these algorithms of metaheuristic nature give approximated solutions of the problem, vital for the moment in which a rapid estimation in big systems is required.

Further, a number of works have shown that optimization problem can be overcome by using Genetic Algorithms (GA). GA are broadly appropriate global search processes based on a stochastic approach. They imitate the process of natural selection, which means those species who are more adapted to changes in their environment are more likely to survive, reproduce and go to the next generation. They stablish a robust search mechanism and contrast from conventional optimization algorithms [14]. GA generate an intelligent exploitation of random search. They have been commonly used to produce high-quality solutions for problems related with structural optimization of steel structures.

Prendes et al. [15] studied the behavior of an elitist GA in order to get the optimization of a three stories structure, in which we define an initial structural setting or zero individual, conformed by sections introduced by the user, therefore, the rest of the individuals are generated randomly, for each generation some individuals die, others mutate finally obtaining an optimal individual bay natural selection. In his article it is not detailed the type of structural system and in consequence the design restrictions considered for the optimization process are not either.

Balogh & Vigh [16] applied GA for the optimal design of regular steel buildings subjected to dynamic loads of earthquakes. Using a parametric study, they tested and calibrated the parameters of the genetic algorithm. They found that GA is able to overall structural as well as bracing layout optimization. The algorithm was developed in Matlab and a simplified structural analysis was incorporated. Furthermore, they used bracing systems with different levels of energy dissipation in their models.

Mujumdar and Matsagar [17] studied the GA technique to optimize a structural member from a beam subjected to a distributed linear load, in his model as a design restriction a relationship not higher than one is imposed among the requests brought to the element and its resistance capacity, resulting in a section with ten percent under the capacity limit. The obtained structural profiles corresponded to normative standards of the place of study, and in its conclusions, future improvements for structures with more elements are clarified.

In the work of Barraza et al. [18], the study of steel structures subjected to such considerations like seism loads was involved, and a comparison between the use of (GAs) and Particles Swarm Optimization (PSO), this last technique, based on social behavior of flocks of birds, insects, among others. In regard to the structural weight PSO obtained a slightly more efficient behavior than GAs, although the type of the employed structural system is not mentioned, neither the computational processing times, in the study it is suggested to consider more objective functions for the design of more complex systems.

Prendes-Gero et al. [19] studied a GA able to work with three different codes: Spanish, European, and American. They defined the objective function based on analyzing constraints and stabilities parameters. Then, they investigated the influence of each parameter over the behavior of the GA for the optimization process. Due to the content coincidences in the building codes, the same genetic algorithm was used for the three cases with a unique modification in the objective function.

Recently, the structural optimization of a Steel arch bridge in Italy was conducted by Feng et al. [20]. They use a GA to face the critical problem of massive horizontal thrust arising in the Calatrava Bridge over the Grand Canal of Venice. They were able to find a reasonably well-defined design. In addition, they get better thickness distributions of steel members and a remarkable reduction of the horizontal thrust. On the other hand, Kumar et al. [21] used several GA to solve the weight minimization problem for a steel truss. They also used binary encoding and applied a discrete optimization to compare the results.

In this context, in the present investigation, a proper technique is developed, in which from the initial condition of a project, a preliminary optimal design of metallic structural profiles that are constructively acceptable is obtained. This one will let the designer save time and direct costs on construction materials. For this, it was necessary to work with data from “W” sections, belonging to the Base of forms AISC v15.0, [22]. Moreover, to show the efficiency of the proposed methodology, a case study was stablished in order to evaluate its optimization processes globally, the same ones that were compared with the obtained results by a specialized tool of commercial use.

## 2 Methodology

### 2.1 Mathematical model

**Figure 1:**
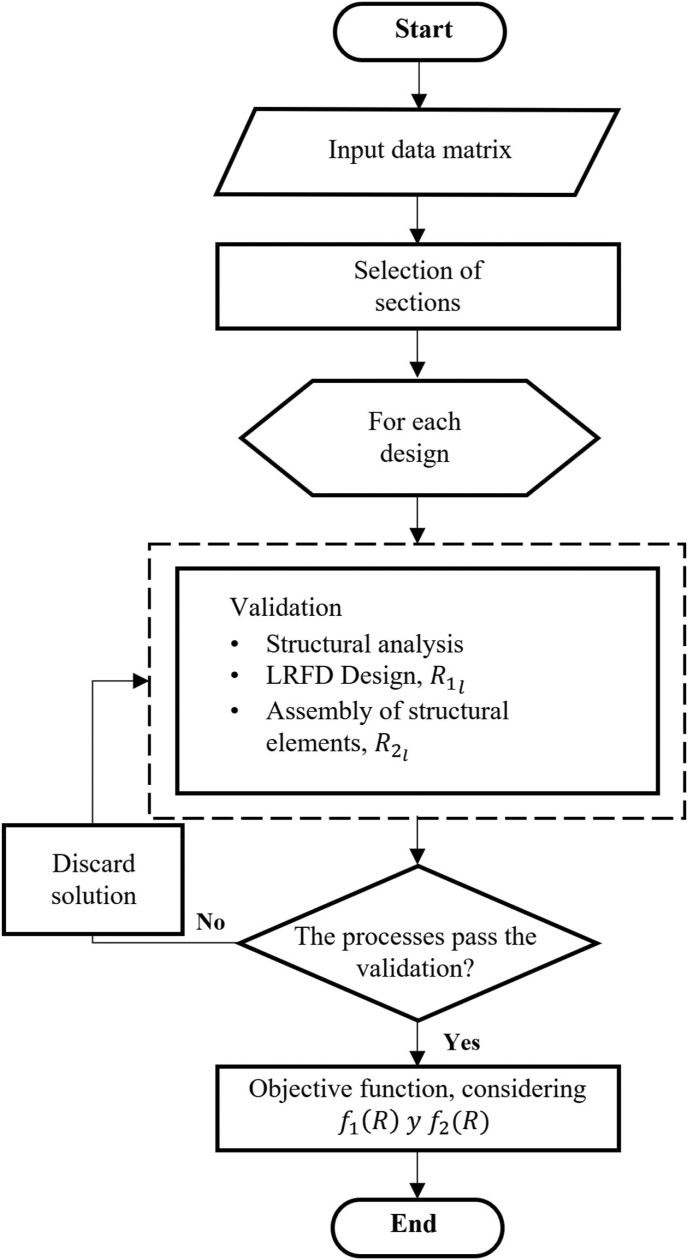
Flow diagram of the performance of the informatic tool.

As it is observed in the flow diagram of (Figure 1), the optimization method shows three processes, which are the following: sections selection, design validation and execution of the objective function.

**Figure 2:**
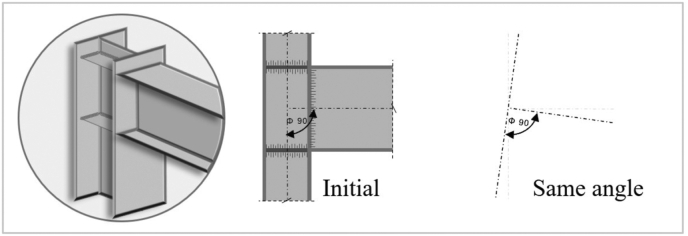
Type of Full Restrained Unit shown on steel elements.

Prior to these processes, information about the project is introduced to the algorithm as a data matrix designed to receive the following parameters: i) nodal coordinates in two dimensions and three degrees of release per node, ii) number of free nodes per frame iii) type of element (beam or column), iv) boundaries conditions and v) loads applied to structural elements. As it is shown in Figure 2, it has been considered to work with a Full Restrained Unit (FR), that is a type of assemble that permits continuity of the wings and the soul through the support element, being able to transmit axial solicitations, cuttings and stages [23].

Then, in order to control computational expenditure, the model starts from a selected group which stablishes the amount and type of sections suitable to define each possible design, and through a procedure of permutations with repetition, in every design a validation process is performed in order to get to the optimal solution in terms of structural weight and constructive complexity. Every process is detailed in the following sections.

### 2.2 Selection of sections

Before describing the method, it is pertinent to explain that every structural model proposed by the user is composed by n structural elements, and for the study the AISC base counts with 274 sections, if search space would consider the whole base, the expression goes:

(1) $$C=274^{n}$$

where C = search space for possible optimal designs.

From these, some can fulfill with the requirements in exchange of being so heavy, meanwhile the others will have lower structural weight before great demands, that is why it results unproductive to look for an optimal design from all this great search space.

For this reason, a selection algorithm will be done, this one will take into consideration analysis criteria of gravitational loads and stress theory on beams and columns, which will permit to stablish ranks that foster the saving of computational expenditure.

In beams the point where the whole transversal section has yielded is defined through an exclusive property of steel, which is known as the plastic section module, Z [24]. The expression can be written as follows

(2) $$\begin{array}{l} Z_{reqi}=\frac{M_{uvi\;}}{\varphi F_{y}},\;i=1,\;\ldots \;,n\\ Cv_{i,j}=\;Z_{reqi}-Z_{j},\;j=1,\;\ldots \;,m \end{array}$$

Where ${Z_{req}}_{i}$ = plastic section module, required for every i-th beam type element; ${M_{uv}}_{i}$ = last moment over every i-th element; φ = reduction factor LRFD; $F_{y}$ = influence stress; $Cv_{i,j}$ = expression which is defines as the coefficient of selection for beam type elements; $Z_{j}$ = plastic section module for each j-th section from the data base of forms AISC; n = number of elements that compose the structure; and m = sections of AISC base.

On their part, in columns it is sought the flaw for inelastic sag, so it is possible to take maximum advantage from the section in stress to flex compression, this procedure can be represented mathematically as follows:

(3) $$\begin{array}{l} \{\begin{array}{c} \varphi P_{nj}=A_{gj}\cdot \varphi F_{crJ}\\ \varphi M_{nj}=Z_{j}\cdot \varphi F_{y} \end{array}\;for\;\frac{kl_{J}}{r_{J}}<4.71\cdot \sqrt{\frac{E}{F_{y}}}\;\\ j=1,\;\ldots \;,m \end{array}$$

where ${P_{n}}_{j}y{M_{n}}_{j}$ = are the nominal resistance to compression and flexion in regard to every j-th section of the AISC base; $\varphi F_{cr}$ = critical stress of sag LRFD in every j-th section of the AISC base; kl/r = slenderness relation (c/t) of the element; and $\sqrt{E/F_{y}}$ = limit slenderness.

Under these considerations, a mathematical expression which defines the interaction between the efforts in the preliminary design stage [25], as it is shown below:

(4) $$Cc_{i,j}=\frac{\sum Q_{i}}{\varphi R_{nj}},\;\begin{array}{l} i=1,\;\ldots \;,n\\ j=1,\;\ldots \;,m \end{array}$$

where $Cc_{i,j}$ expression which is defined as the coefficient for the selection of column type elements; $\sum Q_{i}$ = are the effects of the load over every i-th element (axial and/or moment); $\varphi {R_{n}}_{j}$ = is the preliminary resistance of the section for every j-th section of the AISC base under analysis.

With this, the section is defined according to the following expressions:

(5) $$\begin{array}{l} B_{v}=\left\{m/m\;\in \;B,\;con\;Cv_{i}=\left[-1,\;1\right]\right\}\\ B_{c}=\left\{m/m\;\in \;B,\;con\;Cc_{i}=\left[-1,\;1\right]\right\} \end{array}$$

where, B = data base of AISC structural profiles; $B_{v}yB_{c}=$ grupo de secciones seleccionadas para elementos a flexión y flexocompresión respectivamente, las mismas cuyos coeficientes de selección deben estar en un rango de [-1, 1].

Ranks focus this search space through criteria described in this section, and permits to save computational expenditure, the expression goes like this:

(6) $$C={\left(B_{v}B_{c}\right)}^{n},\;\left(B_{v}\cdot B_{c}\right)\ll 274$$

To stablish the permutation, and additional aspect must be taken into consideration referring the constructive complexity under the next point of view: every level of a typical frame has its own configuration of live and dead loads, which can be the same for all or not. With this coefficient n is modified, since the most practical in constructive terms is to keep a similar beam or column section for each k stories that could exist, therefore:

(7) k≥1,k∈N

Finally, from expression 5 and 6, we get:

(8) C=P(Bv·Bc)k=(Bv·Bc)k

where C = maximum possible value or search space focused for all possible designs.

### 2.3 Design validation

**Figure 3:**
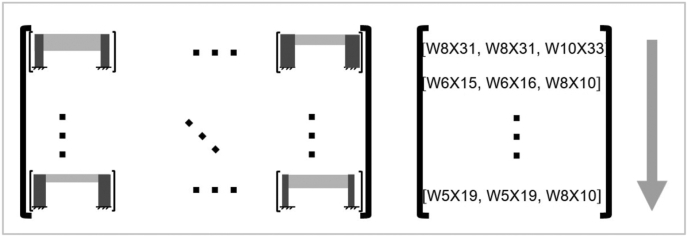
Validation process of steel structural frames, on the left: the possible designs matrix, on the right: validated solutions vector, flow from top to bottom. Notice that in this case it is a 3 elements structural model.

It consists on the evaluation of each generated structural frame, whereby, for every possible solution defined by the previously described procedure, analysis, structural design and assemble verifications will be executed in each node of the structure. Figure 3 is illustrative and shows in a very general way the validation process of the steel structural frames.

As in the present investigation the main topic is flat frame models, the process for obtaining internal strengths is developed through matrix analysis method or rigidity method, which consists on dividing the structural frame into line type elements which have properties of geometrical and mechanical type; regarding purpose, it is very similar to the finite element method, and if we are dealing with bidimensional models, in practical implications with both methods results will be the same [26].

In regard to design restrictions, such standards are based on normative considerations from specification ANSI/ASC 360–16 [27], which rely on conditions of applicable limit state, nominal resistances of each element are related with a resistance factor (φ) which according to the philosophy of LRFD design, corresponds to a value of 0.9 whether for members under compression or flexion. It is necessary to highlight that, for elements under compression effective longitude “k” factors are considered according to the Direct Analysis Method (DAM), a conservative method incorporated from 2010 version in the ANSI/AISC specifications [28]. Reason for which in this project a value of k = 1 is used in any case.

Then, if it is considered the interaction between axials and moments in the structural elements with doubly symmetric sections, the restriction on the design resistance required by a system of Ordinary Moment Frames (OMF) of structural steel goes like this:

(9) $$R_{1l}=\left\{ \begin{array}{ll} \frac{P_{u}}{2\cdot \varphi P_{n}}+\frac{M_{u}}{\varphi M_{n}}\leq 1 & if\frac{P_{u}}{\varphi P_{n}}<0.2\\ \frac{P_{u}}{\varphi P_{n}}+\frac{8}{9}\cdot \frac{M_{u}}{\varphi M_{n}}\leq 1 & if\;\frac{P_{u}}{\varphi P_{n}}\geq 0.2 \end{array}\right.$$

where ${D_{1}}_{l}$ = restriction of LRFD design resistance for the l-th structural frame.

**Figure 4:**
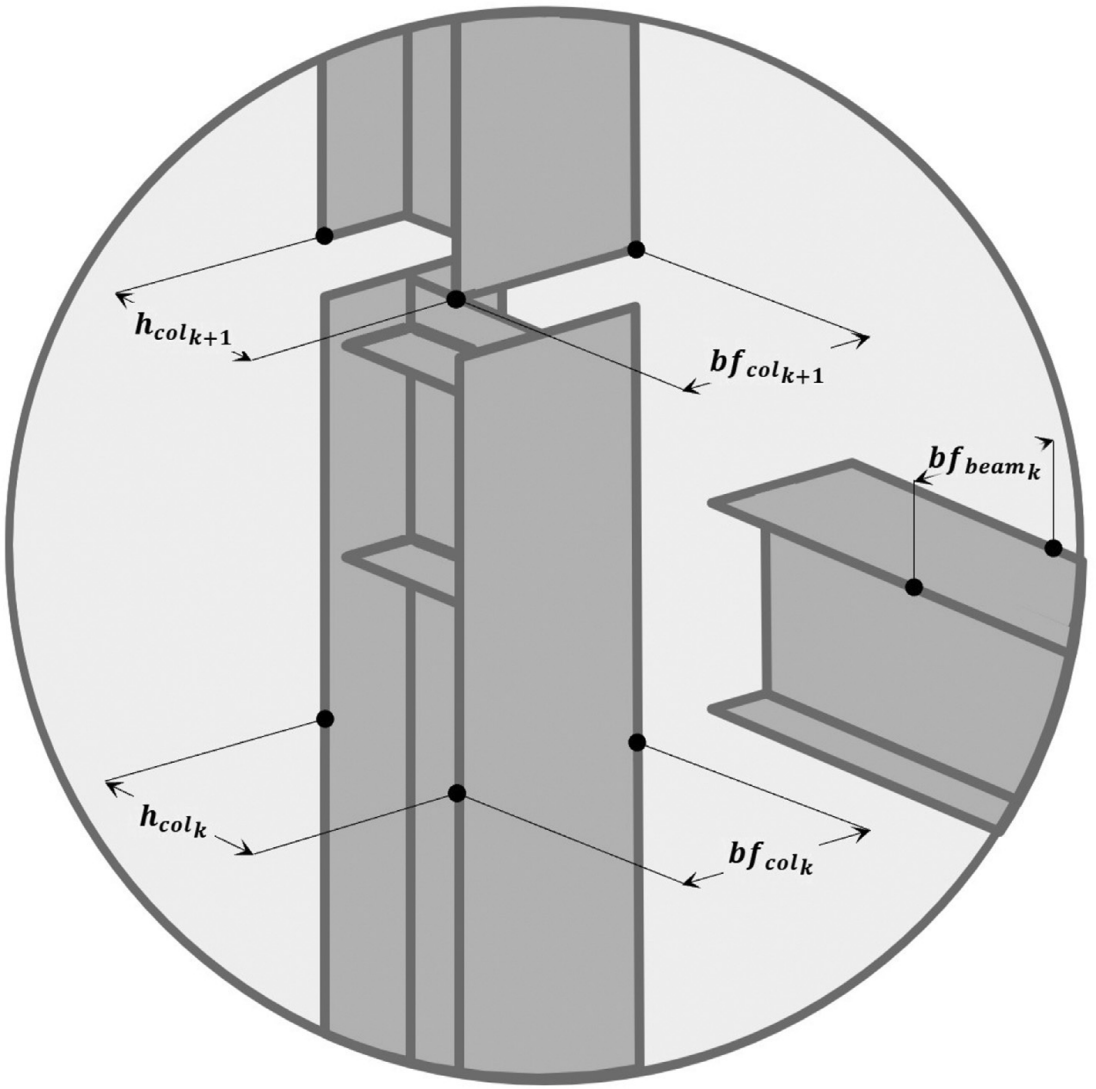
Type of rigid joint illustrated in Steel elements.

Another topic of importance in the preliminary design of structural elements is to satisfy any demand of practical character in the construction, in regard with full restrained joints, a very important aspect is to verify the assemble in every node of the structure beam – column or column – beam. Then, the restriction can be written as it was defined by Gholizadeh and Mohammadi [13]:

(10) $${R_{2}}_{l}=\left\{ \begin{array}{l} \frac{{h_{col}}_{k+1}}{{h_{col}}_{k}}-1\leq 0\\ \frac{b{f_{col}}_{k+1}}{b{f_{col}}_{k}}-1\leq 0\\ \frac{b{f_{vig}}_{k}}{b{f_{col}}_{k}}-1\leq 0 \end{array}\right.$$

where ${R_{2}}_{l}$ = assemble restriction in very node for every l-th structural frame; $h_{col}\;$ = Web of section in column type elements; $bf_{col},\;bf_{vig}$ = skid of section in column and beam type elements respectively; y k = story or level of the structural frame, these variables are detailed in Figure 4.

### 2.4 Objective function

In optimization processes for structural steel frames, there can be more than one conflicted objective to be considered by the designer [29], therefore in this investigation two constraints are evaluated, both of them under the same conditions: the first constraint $f_{1}\left(R\right)$ pretends to minimize the amount of sections or different profiles used in the structural model, which implies to reduce the constructive complexity; and the second constraint possibly more important $f_{2}\left(R\right)$, intends to minimize the weight of the structure and so take maximum advantage of the structural steel sections lowering the weight per linear meter and consequently costs. Having more than one available approach helps offering the designer making a better decision.

The first constraint can be expressed the following way:

(11) $$Minimizar:\;f_{1}\left(R\right)+=\frac{S_{i}}{n}\;if\;S_{i}\neq S_{i-1},\;i=1,\;\ldots \;,n\;$$

where, $f_{1}\left(R\right)$ = shows constructive complexity, notice that the value cannot be more than 1, in every case this value means that all the elements of the steel frame have different sections; and $S_{i}$ = evaluated section in the i-th element.

The second constraint can be formulated through the next mathematical expression:

(12) $$Minimizar:\;f_{2}\left(R\right)=\sum_{i=1}^{n}w_{i}\cdot L_{i}$$

where, $f_{2}\left(R\right)$ = frame's net weight; R = group of restrictions by which the function is subjected, see Eq. (8); $w_{i}$ = weight per longitude unit of every i-th assigned section in the structural frame; and $L_{i}$ = longitude of every i-th element of the structural frame.

### 2.5 Descriptive environment of the software

**Figure 5:**
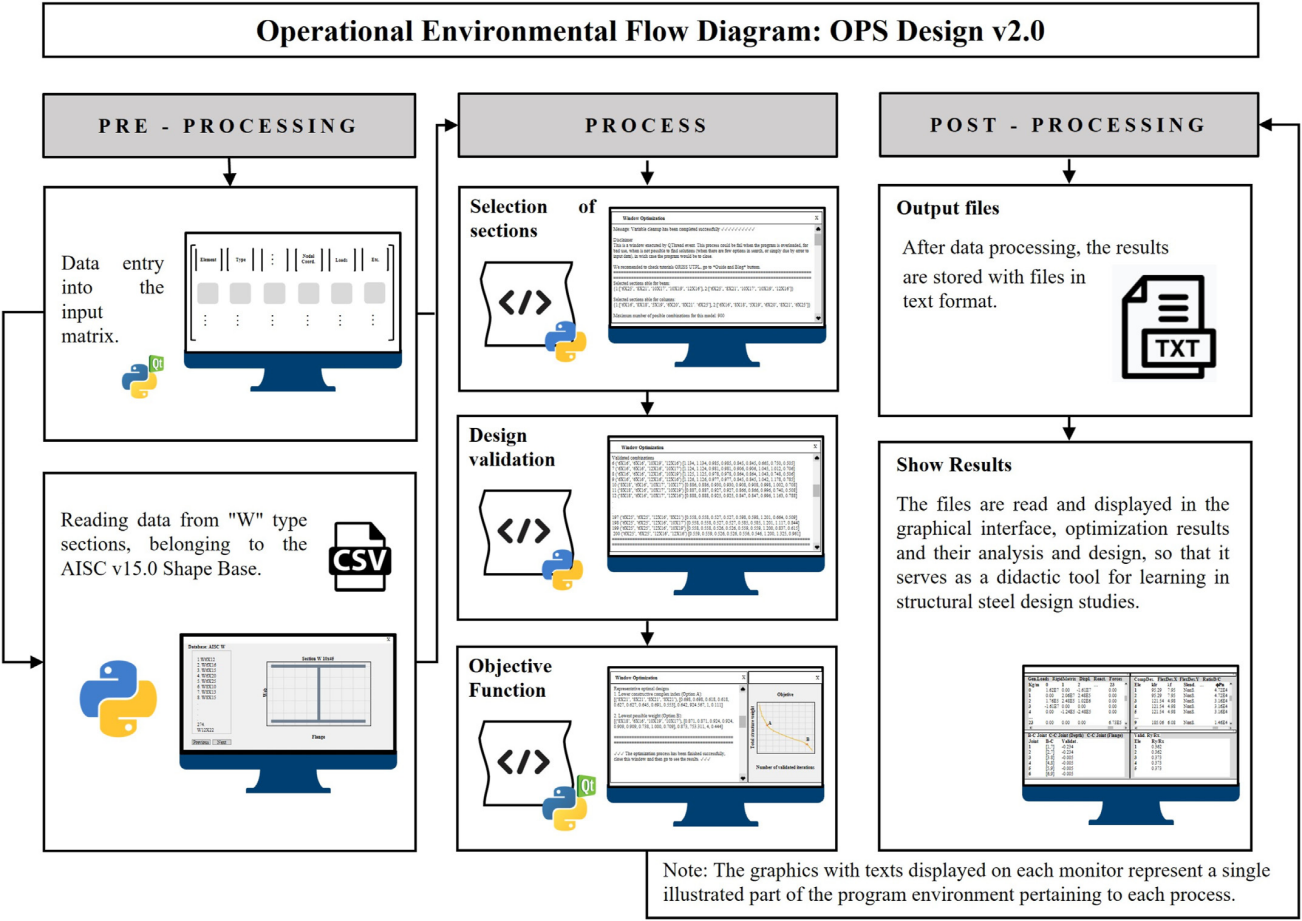
Diagram of operational flow of the graphic environment of OPS Design v2.0.

The development of the application denominated OPS Design v2.0, has been developed in the platform Python 3.7.4, as it can be seen in Figure 5, its performance shows three stages: in the first stage we have the pre-process, in which the visualized data is entered through a graphic interface developed in the framework of PyQt5; in the second stage data is processed, here the section selection procedure is executed, as long as design and objective function validation, explained in previous sections; and finally the third stage, in which the information post-process happens, here the results of the most optimal designs are stored in files generated by the application to be read in the interface.

The source code as well as the user's manual have been located in GitHub repository for public domain (https://github.com/grissutpl/OPS-Design.git).

**Figure 6:**
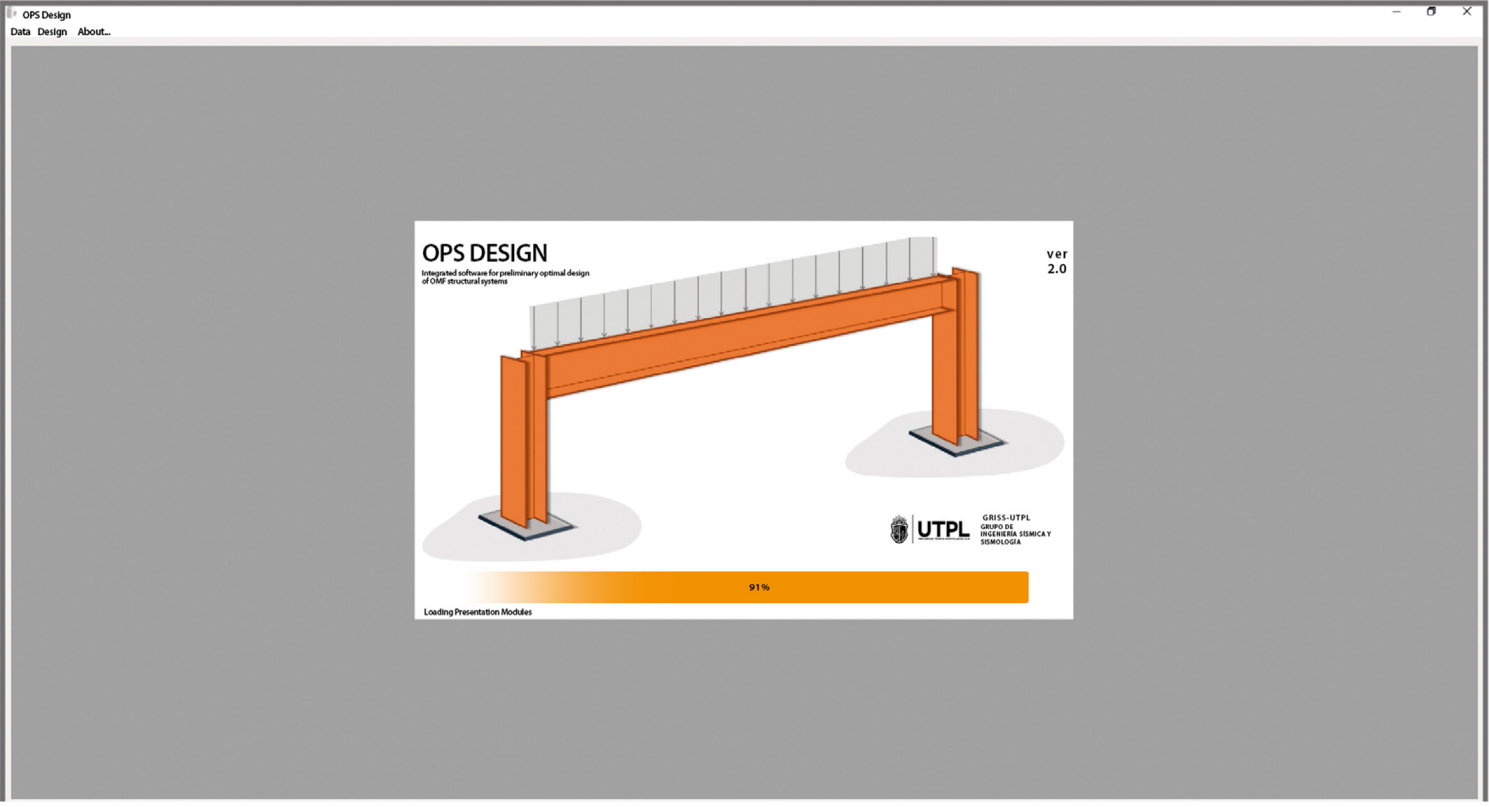
Main window.

**Figure 7:**
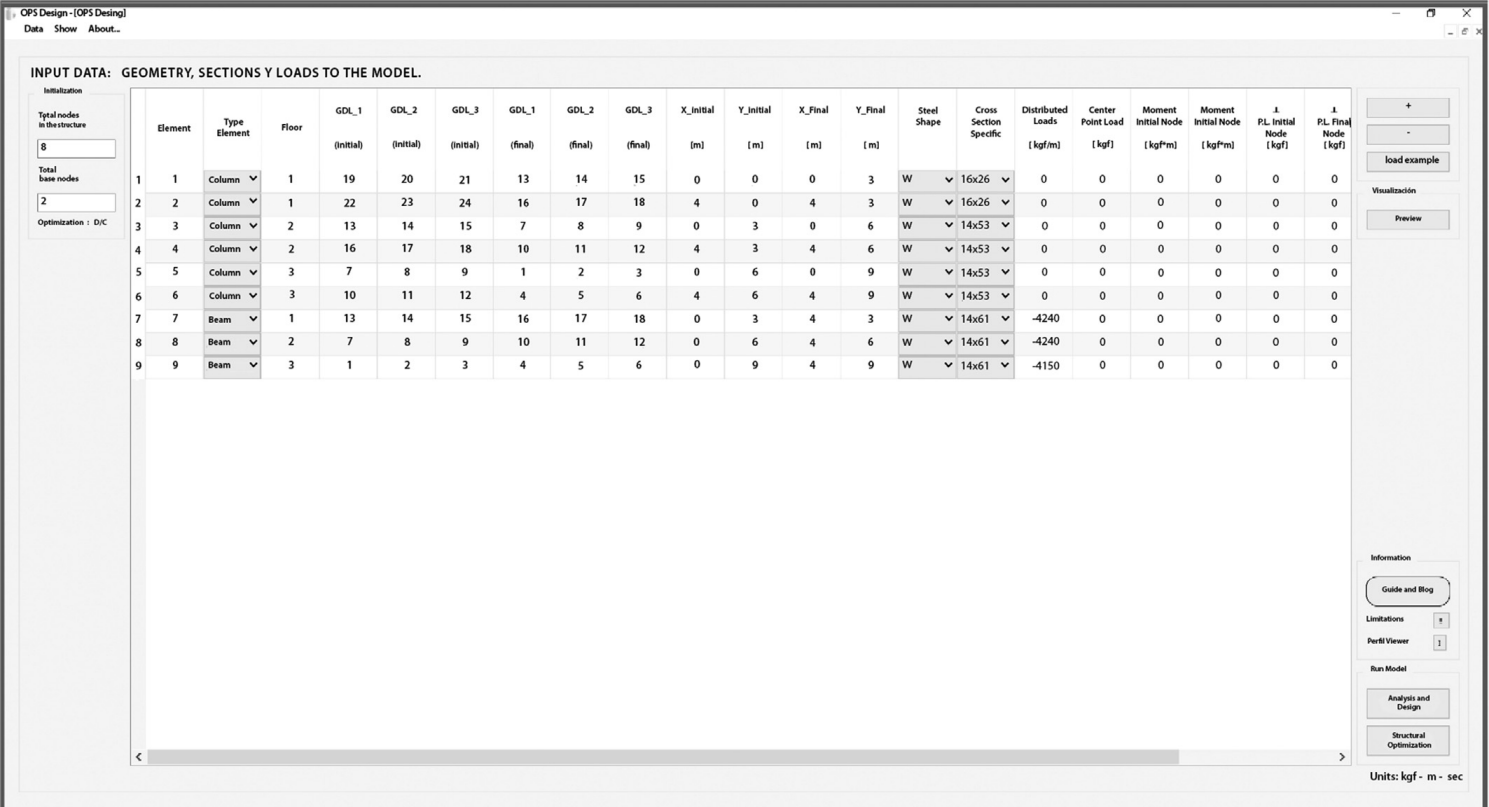
Data entry sub-window.

When starting the software, a start window will show (see Figure 6) which has a menu bar at the upper part. Through this we get access to a sub-window of data entry in which the necessary steel frame parameters must be registered (see Figure 7).

**Figure 8:**
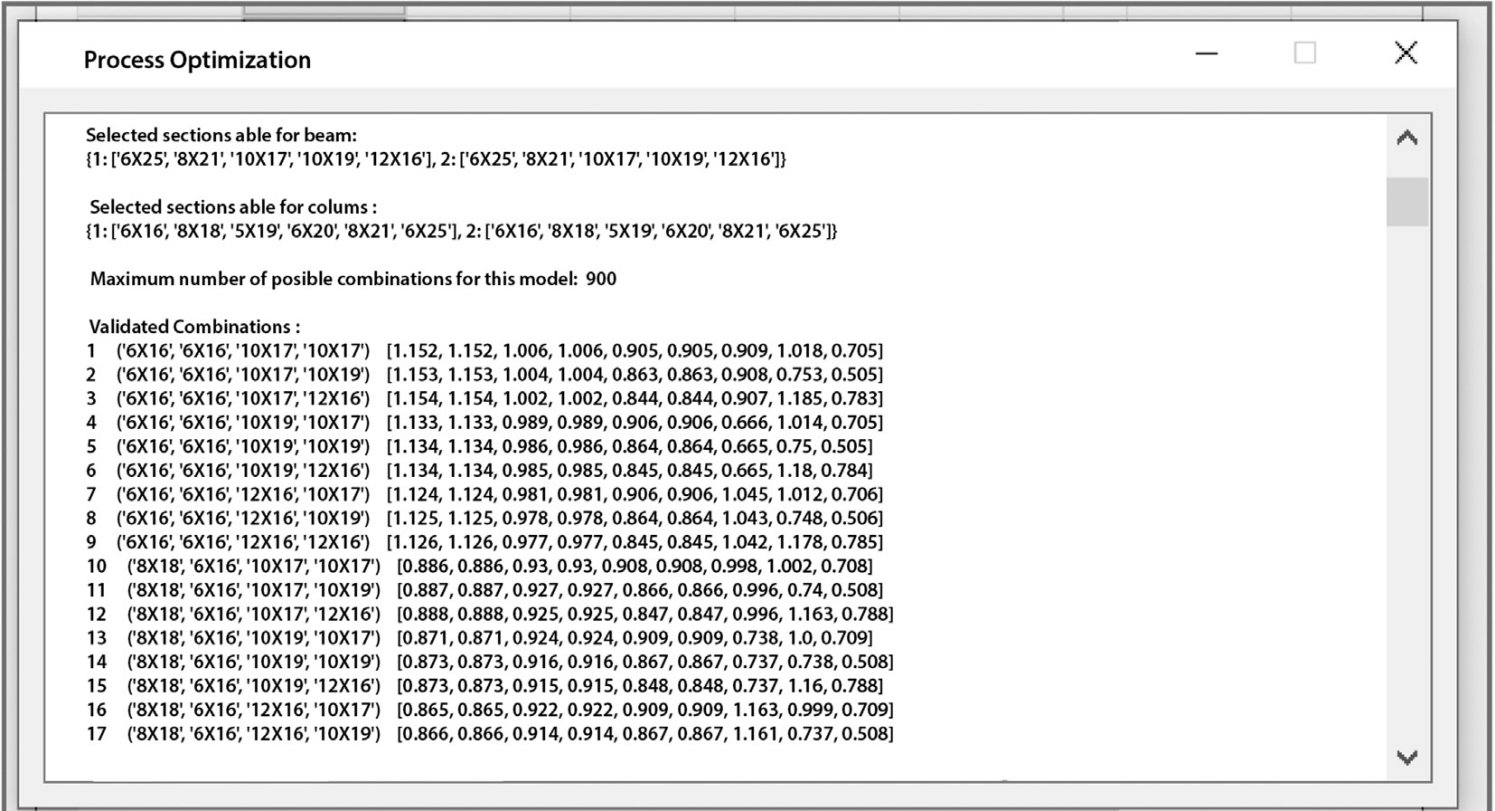
Optimization window.

Once all the data has been registered the next step is the execution of the tool, at the same time a window will automatically pop-up with the optimization procedure in real time, which shows on each screen all the possible designs that have been performed with the families of selected structural profiles until it gets the most optimal structural options (see Figure 8).

**Figure 9:**
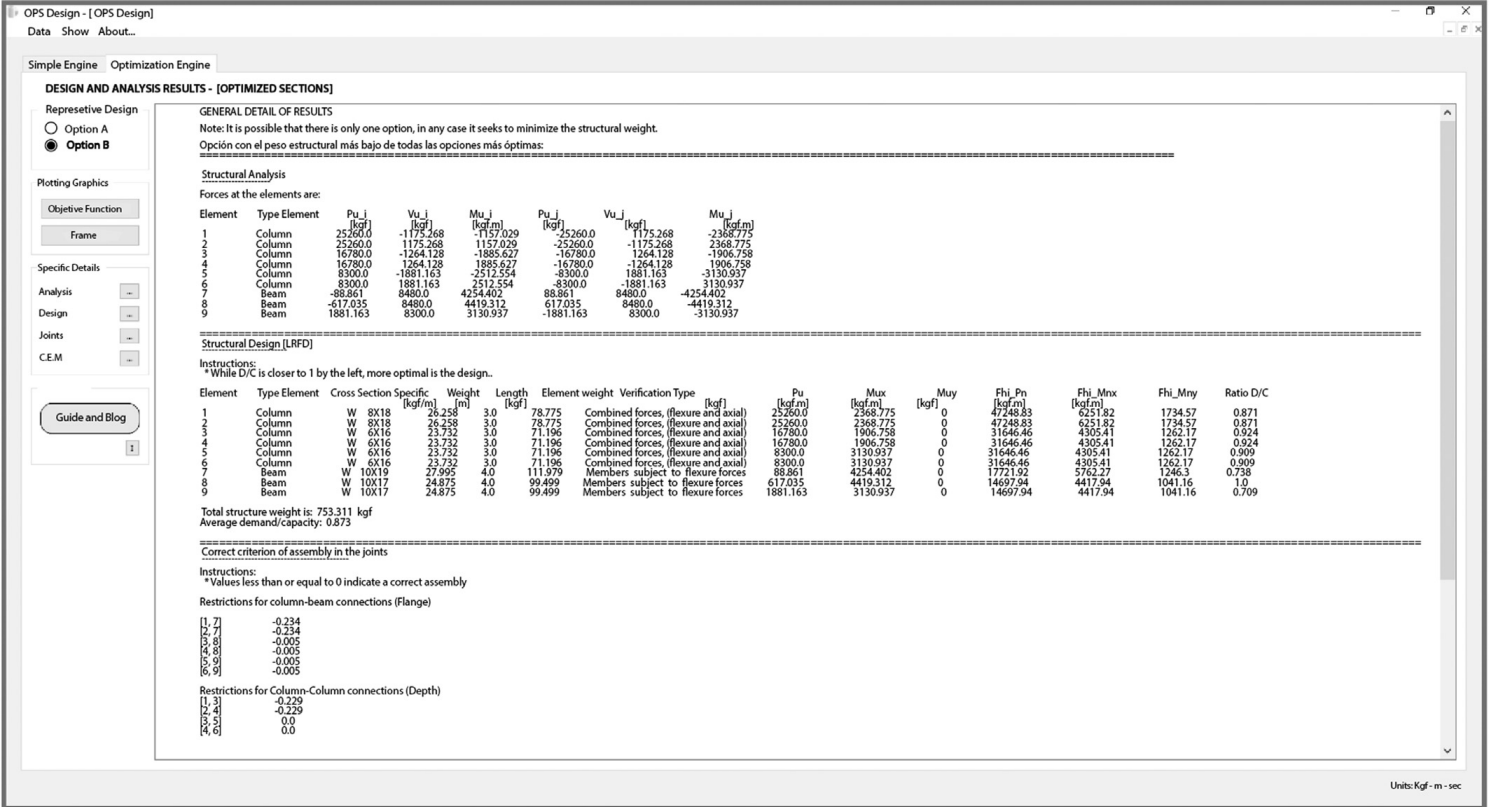
Window of general results.

Once finished the procedure of the optimization window, the program lets visualize the results in a detailed way, among them: display of graphics like the relation structural weight with number of repetitions, detail of the frame and used sections, as well as specific of analysis, design and constructive details that can be viewed in charts (see Figure 9).

The software also presents a window od assistance which briefly describes the functions of the tool in its first version release. A technical sheet of the software is also available in the Web page of the Research Group in Seismic Engineering and Seismology of the Technical University of Loja (https://ingenieriasismica.utpl.edu.ec/?q=es/OPS_Design).

### 2.6 Representative illustration

**Figure 10:**
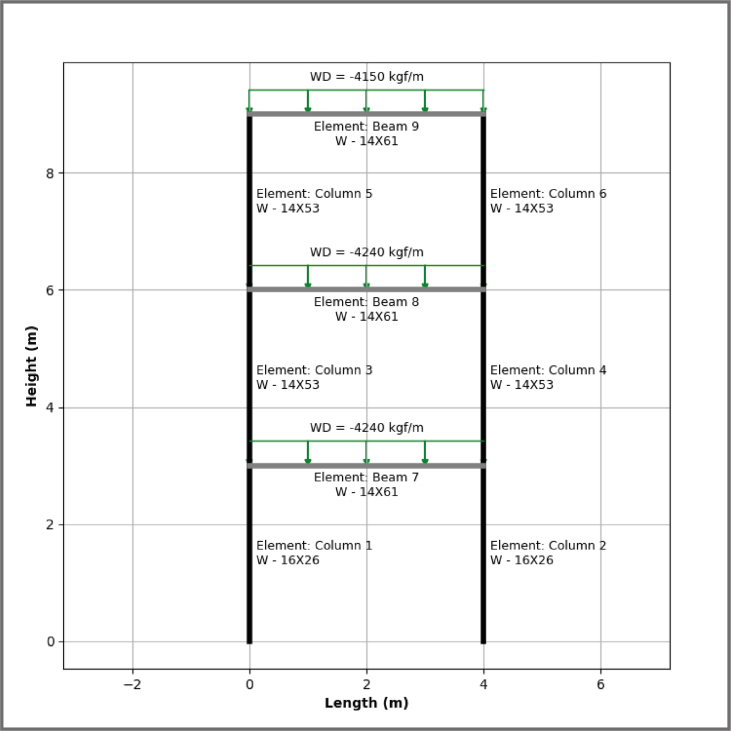
Preview of 2D model.

To evaluate the optimization processes the proposed tool has, in Figure 10 a case study of a flat steel frame denominated ‘M1A_3P’, which has the next characteristics: number of stories: 3; number of compartments: 1; load of design in the first two stories of: 4240 kgf/m and for the last story 4150 kgf/m; mezzanine height: 3.0 m; and longitude of the compartment: 4.0 m; the used material is steel ASTM degree 36.

For the evaluation a 2.8 GHz CPU is used, the execution times are measured in function of the equipment performance.

**Figure 11:**
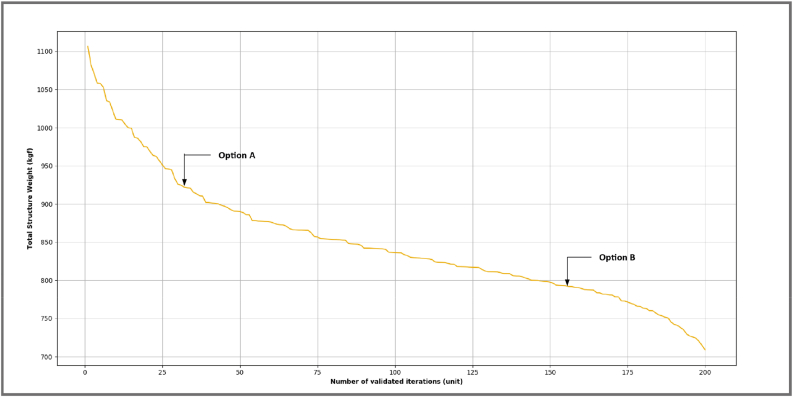
Curve of the structural weight for every valid option.

Through the use of the algorithm of selection proposed in the optimization model, for this example a search place was generated, it was defined by a total of 900 possible solutions of steel structural frames. Then, by means of the algorithm of validation the restrictions defined for every generated structural set up were checked up, resulting on a list of 200 optimal design options. The selected group of optimal solutions forms a curve of the weight of the structure vs the corresponding optimal options (validated), see Figure 11.Wherewith, through the constraints described before minimizing constructive complexity and structural weight, two representative optimal designs are obtained.

Design option A represents the minimum number of sections or different profiles for the proposed structural model, this implies a better constructive complexity; and design option B represents the minimum structural weight (cost) for such structural model, both options fulfill the design restrictions previously described. It should be mentioned there are several structural optimizations works undertaken even on commercial tools, Table 1 shows in detail the results of the undertaken optimization by using the proposed tool along with an optimal design developed with the features of a commercial tool.

**Table 1:** Detail of representative designs along with the one of the commercial tools.

Element	Design A		Design B		Commercial Tool	
	Profiles	Ratio D/C	Profiles	Ratio D/C	Profiles	Ratio D/C
1	W 8 × 21	0.698	W 8 × 18	0.871	W 8 × 18	0.822
2	W 8 × 21	0.698	W 8 × 18	0.871	W 8 × 18	0.822
3	W 8 × 21	0.618	W 6 × 16	0.924	W 6 × 15	0.933
4	W 8 × 21	0.618	W 6 × 16	0.924	W 6 × 15	0.933
5	W 8 × 21	0.627	W 6 × 16	0.909	W 12 × 16	0.847
6	W 8 × 21	0.627	W 6 × 16	0.909	W 12 × 16	0.847
7	W 8 × 21	0.645	W 10 × 17	0.738	W 8 × 18	0.811
8	W 8 × 21	0.691	W 10 × 19	1.000	W 8 × 18	0.786
9	W 8 × 21	0.553	W 10 × 19	0.709	W 8 × 18	0.890
Average Capacity Demand		0.642		0.873		0.855
Structural Weight		924.570		765.910		746.430
Number of Profiles		1.000		4.000		3.000
Constructive Complexity		0.11		0.44		0.33

For every design, the slenderness restriction in elements under compression is shown in Table 2. Verifications of beams and columns assembles for every node and under the proposed model are shown in Tables 3 and 4 respectively.

**Table 2:** Detail of representative designs along with the one of the commercial tools.

Element	Type of Element	Design A	Design B	Commercial Tool
		klr_Design	klr_Design	klr_Design
1	Column	93.23	95.29	96.77
2	Column	93.23	95.29	96.77
3	Column	93.23	121.54	81.08
4	Column	93.23	121.54	81.08
5	Column	93.23	121.54	150
6	Column	93.23	121.54	150

**Table 3:** Detailed information of beam – column assembles.

Joint Beam - Column	Design A	Design B	Commercial Tool
[1, 7]	0	-0.236	0
[2, 7]	0	-0.236	0
[3, 8]	0	-0.002	-0.124
[4, 8]	0	-0.002	-0.124
[5, 9]	0	-0.002	0.316
[6, 9]	0	-0.002	0.316

**Table 4:** Detailed information of beam – column assembles.

Joint Column - Column	Design A		Design B		Commercial Tool	
	Flange	Web	Flange	Web	Flange	Web
[1, 3]	0	0	-0.229	-0.232	0.141	-0.264
[2, 4]	0	0	-0.229	-0.232	0.141	-0.264
[3, 5]	0	0	0	0	-0.334	1.003
[4, 6]	0	0	0	0	-0.334	1.003

Tables 3 and 4, presents a list that shows the two elements assembled to the frame, and to the right the respective validation coefficients; zero and negative values show a correct assemble.

## 3 Results

With a total of 900 possible solutions, and a total computational time of performance of the optimization process of 50.0 s, can be considered an acceptable computational effort. As it highlights [30] a good optimization process must be efficient and with consistent solutions. For that matter, the limits defined in the coefficients $Cv_{i}$ y $Cc_{i}$ from the algorithm of selection permit to focus the search space by discretizing through the theory of efforts the number of sections of the AISC base in order to reach a number of privileged sections for the permutation process. Since the objective is to minimize structural weight and constructive complexity, two results options that evaluate such constraints are presented. Option A throws a structural weight of 924.57 kg with 0.11 of constructive complexity, on the other hand option B throws a structural weight of 765.91 kg with 0.44 of constructive complexity, while the commercial tool throws a weight of 746.43 kg and 0.33 of constructive complexity.

For that matter, design A is the optimal one when the purpose is to minimize the number of different profiles or constructive complexity, while design B is the optimal one when the purpose is to minimize structural weight, all these designs fulfill with the design restrictions of this study. On the other hand, the design of the commercial tool does not fulfill with all the restrictions define for the level of ordinary frame design in non-braced systems.

In regard of the previous paragraph, it can be observed in Table 2 that for designs A and B of the proposed tool, the slenderness relationships do not reach the limit, they are not even close to be considered short columns, so all their elements under compression have a failure for inelastic sag, using this way their profiles, something than does not occur with columns in the design of the commercial tool.

Regarding the beam and column assembles, in Tables 3 and 4 can be observed that for designs A and B there are zero and negative values, which shows a correct assemble in each rigid joint node, while in designs of commercial tools the restriction is not fulfilled.

At this point, it can be inferred that the selection of an optimal design alternative depends on some factors, optimizing implies evaluating the case study presented from a global point of view, therefore, every design alternative presented for the proposed tool as well as the commercial tool can have superior characteristics compared to each other; in Table 5 a matrix is shown with the characteristics of every design presented by both tools, the multicriteria analysis of theses results is made through the normalized weighted method, it must be explained thar the informatic tool does not incorporate the multicriteria analysis.

**Table 5:** Summary of characteristics for every presented design.

Methods	D/C [Media]	Assemble in joints	Sag Restriction form	Number of Profiles	Structural Weight
	**P1**	**P1**	**P1**	**P3**	**P2**
Design A	0.642	10.000	6.000	1.000	924.570
Design B	0.873	10.000	6.000	4.000	765.910
Commercial Tool	0.855	6.000	4.000	3.000	746.430
Maximum	0.873	10.000	6.000	4.000	924.570
Minimum	0.642	6.000	4.000	1.000	746.430
Range	0.231	4.000	2.000	3.000	178.140

The matrix of normalization of characteristics is presented in a subsequent way in Table 6, in this method the lower value determines the best option having as referent the characteristics and its priorities defined in the previous table. Therefore, the best option represents design B from the informatic tool proposed in this investigation.

**Table 6:** Matrix of normalization of characteristics.

Criteria	Weights	Design A		Design B		Commercial Tool	
		Charact.	Score	Charact.	Score	Charact.	Score
D/C	0.136	0.642	1.000	0.873	0.000	0.855	0.078
Joints	0.136	10.000	0.000	10.000	0.000	6.000	1.000
Sag	0.136	6.000	0.000	6.000	0.000	4.000	1.000
Number	0.180	1.000	0.000	4.000	1.000	3.000	0.667
Structural Weight	0.273	924.570	1.000	765.910	0.109	746.430	0.000
Total	**1.000**	**Total**	**0.409**	**Total**	**0.210**	**Total**	**0.403**

## 4 Conclusions

This paper presents OPS Design v2.0, informatic tool developed in Python which users graphic interface permits the obtention of optimal designs of ordinary moment frames in an easy and intuitive way, highlighting the following aspects:

Search spaces determined by the proposed tool for the optimization result effective for the reduction of computational expenses, since it discretizes the group of AISC sections, taking into consideration analysis criteria of gravitational loads and stress theory.

For the evaluation of every structural frame, we fulfill simultaneously with restrictive variables of design that include resistance of its structural elements, the performance of members under compression through its sag ways, and restrictive variables of constructive character that include the assemble of every node in the structure. As a result, the design options that re obtained show improvements in correspondence to other optimization methods that are employed even in tools of commercial use.

The performance of the proposed tool compared to a commercial one, was evaluated through a case study. The results of the optimal design show the efficiency of the proposed tool and show a better design option in comparison with the commercial tool, fulfilling in this way with all the defined restriction for the methodology. With this, the model contributes to the reduction of employed time for the designer in usual iterative processes that are usually performed during the initial stages of the project.

## Declarations

### Author contribution statement

Edwin P. Duque: Analyzed and interpreted the data; Wrote the paper.

Daniel Villarreal: Performed the experiments; Wrote the paper.

Henrry Rojas A: Conceived and designed the experiments; Analyzed and interpreted the data.

### Funding statement

This research did not receive any specific grant from funding agencies in the public, commercial, or not-for-profit sectors.

### Data availability statement

No data was used for the research described in the article.

### Declaration of interests statement

The authors declare no conflict of interest.

### Additional information

No additional information is available for this paper.

## Appendix A Supplementary data

ImagesElsevierHelyonEnglish2.0 ImagesElsevierHelyonEnglish2.0
